# Phylogenetics, Molecular Species Delimitation and Geometric Morphometrics of All Reddish-Brown Species in the Genus *Neotriplax* Lewis, 1887 (Coleoptera: Erotylidae: Tritomini) †

**DOI:** 10.3390/insects15070508

**Published:** 2024-07-06

**Authors:** Jing Liu, Huixin Xu, Ziqing Wang, Panpan Li, Zihan Yan, Ming Bai, Jing Li

**Affiliations:** 1 College of Plant Protection, Hebei Agricultural University, Baoding 071000, China; liujing15231147432@126.com (J.L.); xu13463110473@163.com (H.X.); 13184942007@163.com (Z.W.); 2 Key Laboratory of Animal Biodiversity Conservation and Integrated Pest Management (Chinese Academy of Sciences), Institute of Zoology, Chinese Academy of Sciences, Beijing 100101, China; lipanpan@st.gxu.edu.cn; 3 Shijiazhuang Center for Disease Control and Prevention, Shijiazhuang 050011, China; yzh192803@163.com

**Keywords:** DNA barcoding, phylogeny, systematics, species delimitation, geometric morphometrics

## Abstract

**Simple Summary:**

Due to the instability of interspecific morphological characteristics and the significant convergence phenomenon, it is difficult to identify most species of *Neotriplax* based on morphology. Through integrated research on the reddish-brown species of *Neotriplax*, which feature many phenotypic similarities, and by comprehensively utilizing morphology, molecular phylogeny, and geometric morphometrics, the relationship among all these species was reconstructed. The taxonomic status within the genus was partly clarified, with three new species being identified: *N. qinghaiensis* sp. nov., *N. maoershanensis* sp. nov., and *N. guangxiensis* sp. nov.

**Abstract:**

To date, five species of reddish-brown *Neotriplax* have been described, but their highly similar body color and other phenotypic traits make accurate taxonomy challenging. To clarify species-level taxonomy and validate potential new species, the cytochrome oxidase subunit I (*COI*) was used for phylogenetic analysis and the geometric morphometrics of elytron, pronotum, and hind wing were employed to distinguish all reddish-brown *Neotriplax* species. Phylogenetic results using maximum likelihood and Bayesian analyses of *COI* sequences aligned well with the current taxonomy of the *Neotriplax* species group. Significant K_2_P divergences, with no overlap between intra- and interspecific genetic distances, were obtained in *Neotriplax* species. The automatic barcode gap discovery (ABGD), assemble species by automatic partitioning (ASAP), and generalized mixed Yule coalescent (GMYC) approaches concurred, dividing the similar species into eight molecular operational taxonomic units (MOTUs). Geometric morphometric analysis using pronotum, elytron, hind wing shape and wing vein patterns also validated the classification of all eight species. By integrating these analytical approaches with morphological evidence, we successfully delineated the reddish-brown species of *Neotriplax* into eight species with three new species: *N. qinghaiensis* sp. nov., *N. maoershanensis* sp. nov., and *N. guangxiensis* sp. nov. Furthermore, we documented the first record of *N. lewisii* in China. This study underscores the utility of an integrative taxonomy approach in species delimitation within *Neotriplax* and serves as a reference for the taxonomic revision of other morphologically challenging beetles through integrative taxonomy.

## 1. Introduction

Erotylinae is the largest subfamily in Erotylidae, encompassing five tribes and more than 2600 species [1,2,3]. The adults and larvae feed on fungi, and some of them are important pests in regard to edible mushrooms [4]. The genus *Neotriplax* belongs to Erotylinae, Tritomini, and was established by Lewis for the species *Neotriplax atrata* Lewis, 1887 [5]. There are nine species of this genus *Neotriplax* (Erotylinae, Tritomini) that are distributed in China, Japan, India, South Korea, Bhutan, Nepal, and Russia [6,7,8].

The color of *Neotriplax atrata* Lewis,1887 and *Neotriplax delkskampi* Nakane,1961 are uniformly black; the color of *Neotriplax pallidicincta* Lewis,1887 is generally black without the lateral margin of elytra yellow, and *Neotriplax biplagiata* Lewis,1887 has red patches in the elytra [7,9]. The body colors of the five remaining species are reddish-brown, including *N. rubens* (Hope, 1831), *N. lewisii* Crotch, 1873, *N. minima* Li and Ren, 2006, *N. arisana* Miwa, 1929 and *N. miwai* Nakane, 1966. These reddish-brown *Neotriplax* are typically distinguished by coxal lines, body size, the aspect ratio of the pronotum, and coloration [9,10,11,12,13,14,15,16].

However, these morphological characteristics have limitations. For example, the length of postmetacoxal lines is used to distinguish between *N. arisana* and *N. miwai*. However, the length of coxal lines varies among different individuals, making it an unstable characteristic; insect size can vary depending on their growing environment [17,18,19]. In our observation, we also found that different preservation methods may lead to color variation in samples. Therefore, relying solely on morphological characteristics for species identification can be challenging. Integrating molecular phylogeny, morphology, and geometric morphometrics complement morphological characteristics for species classification [20,21,22,23].

DNA-based taxonomy has promoted the molecular identification and discovery of cryptic species [24,25,26,27,28,29]. The use of DNA barcoding increased the number of species in different taxa [30,31,32,33]. DNA barcoding regions have been widely used in recognizing species boundaries [34,35,36,37,38,39,40]. The ABGD algorithm is mainly based on the characteristics of the distance distribution between DNA sequences to find the blank areas of DNA barcodes and carry out MOTUs [34]. ASAP (assemble species by automatic partitioning) uses hierarchical clustering algorithms to classify species by calculating pairwise genetic distances [41]. GMYC combines the Yule model of speciation with the neutral coalescence model of intraspecific branching to provide a robust approach to define species boundaries [35]. The bPTP method calculates these two values by assuming that the number of substitutions within a species is lower than the number of substitutions between species for the purpose of distinguishing species [36].

Geometric morphometrics enables statistically comparing of morphological characteristic differences through quantitative analysis [42,43,44]. Compared with traditional methods, geometric morphometrics significantly reduces the potential error in morphological assessments by avoiding the subjective judgement of taxonomists and enabling them to have more complete evidence for statistical operations [45]. Therefore, it plays an increasingly important role in the distinction of closely related genera and species, the discovery of cryptic species, and the identification of subspecies [46,47].

This study analyzed the *COI* gene of reddish-brown *Neotriplax* species and calculated the intraspecific genetic distance (intra-GD) and interspecific genetic distance (inter-GD). ABGD, ASAP, GMYC, and bPTP were used to identify molecular species boundaries. A geometric morphometric method was used quantitatively to analyze and compare the shape variation in the pronotum, elytron and hind wing of the similar species of *Neotriplax*. This is the first integrated taxonomic study of the genus, providing novel insights and approaches for the classification of *Neotriplax*. By integrating the data from morphology, molecular markers, and quantitative morphometrics, the reddish-brown species of *Neotriplax* was classified as eight species in the world.

## 2. Materials and Methods

### 2.1. Specimen Collection and Preservation

All specimen information for this study is provided in Table S1. The specimens used in molecular systematics studies were stored in ethyl alcohol at −20 °C. Additionally, some dried specimens were examined for geometric morphometric studies. All type species are currently deposited at the Museum of Hebei University (MHBU), the Institute of Zoology, the Chinese Academy of Sciences (IZAS), and the Hebei Agricultural University (HEBAU).

### 2.2. Morphological Comparison and Terminology

Habitus photographs of specimens were captured using the Olympus E-M5II camera (OLYMPUS, Beijing, China) for stacked photography. The detailed photographs of mouthparts and genitalia were illustrated using an α7RIII SONY camera (SONY, Tokyo, Japan). Final plates were enhanced using Adobe Photoshop CS6.0 (Adobe Systems Inc., San José, CA, USA). Morphological terminology follows Lawrence et al. [48,49].

### 2.3. Genomic DNA Extraction, Sequence Amplification and Data Analysis

A DNeasy Blood & Tissue kit (TIANGEN, Beijing, China) was used to extract the isolated genomic DNA. DNA quality and concentration were measured on the Nanodrop2000 (ND-2000) spectrophotometer (Thermo fisher, USA) and assessed through electrophoresis in a 1% agarose gel.

A fragment of the *COI* gene has been proven to be a standard DNA barcode region for animal species identification and the detecting of molecular operational taxonomic units (MOTUs) [50], which can be widely used in different fields (for example, species delimitation [51] and cryptic diversity discovering [52]). The primers used to amplify mt DNA *COI* were synthesized by the Biomed company (Beijing, China) and are listed in Table S2 [53,54]. The *COI* was amplified using the 40 μL system: 2×Taq PCR Mix 20 μL, forward and reverse primer 1 μL, template DNA 2 μL, ddH_2_O 16μL. The reaction procedure at the system of *COI* was pre-denaturation at 94 °C for 4 mins, denaturation at 94 °C for 30 s, annealing at 52 °C for 1 min, extension at 72 °C for 1 min, 35 cycles and extension at 72 °C for 7 min. The sequencing results were spliced and corrected using Chromas v2.6.6. A Nucleotide BLAST on the NCBI (https://www.ncbi.nlm.nih.gov/) (accessed on 1 Jun 2023) was used to test the accuracy of sequence.

Clustal_X [55] was used to conduct DNA alignment from the amino acid alignment of *COI*. *Dacne picta* (Erotylidae: Dacnini) was selected as outgroup [56]. The maximun likelihood (ML) method was conducted with IQ-Tree v1.6.8 in Phylosuite to construct the phylogenetic tree [57,58]. The TIM2 + F + I + G4 model was selected by ModelFinder. A model of nucleotide evolution was selected using the Akaike Information Criterion (AIC) [59]. Branch support was evaluated using the ultra-fast bootstrapping method with 1000 replicates [60]. Phylogenetic analysis was conducted using MrBayes v3.2, run with a total of 2 million generations, sampled every 100 generations, and cut off with 20% of the sampled trees [61]. Two Markov chain Monte Carlo (MCMC) chains were employed on DNA sequence alignment with the GTR + F + I + G4 model. FigTree v1.4.3 [62] was used to view and illustrate the inferred phylogenetic trees. A Kimura 2-parameter (K_2_P) genetic distance model was used to calculate the intra- and interspecific genetic divergences [63,64]. One way analysis of variance (ANOVA) and LSD multiple comparison were used to detect pairwise differences.

ABGD was performed on a web interface (https://bioinfo.mnhn.fr/abi/public/abgd/abgdweb.html) (accessed on 24 May 2024) with a K_2_P model to classify different species based on genetic distance [34]. The value of relative gap width (X) was set as 1.0, prior intraspecific divergence (P) was set as 0.001–0.1, and steps was set as 20 [65]. ASAP (https://bioinfo.mnhn.fr/abi/public/asap/asapweb.html) (accessed on 24 May 2024) was used with the K_2_P model [41]. GMYC: An ultrametric tree was produced using the BEAST v1.10.4 [66]. The settings were as follows: MCMC chain using 200 million generations, GTR + I + G4 model, strict clock, and coalescent: constant size, log parameters every 20,000 generations. The default Burn-In value was 10%. Tracer v1.7.1 [67] was used to evaluate the convergence of the system Tree, and Tree Annotator v1.10.4 was used to generate the ultrametric tree. The ultrametric tree was delimited with the SPLITS package (http://r-forge.rproject.org/projects/splits/) (accessed on 25 May 2024) using the R program [68]. For bPTP, a maximum likelihood (ML) tree was used in the bPTP web server (http://species.h-its.org/ptp/) (accessed on 25 May 2024), setting to a rooted tree, MCMC generations: 200,000. All sequenced fragments were submitted to GenBank (https://www.ncbi.nlm.nih.gov/) (accessed on 5 Mar 2024), accession numbers see Table S1).

### 2.4. Geometric Morphometrics Analysis

An Olympus E-M5II camera (OLYMPUS, Beijing, China) was utilized to capture two-dimensional images of the pronotum, elytron and wing. To ensure accurate geometric morphometric analysis, it was crucial to maintain consistent placement position and shooting orientation throughout the photography process. The specimens used for geometric morphometrics are shown in Table S1.

**Figure 1:**
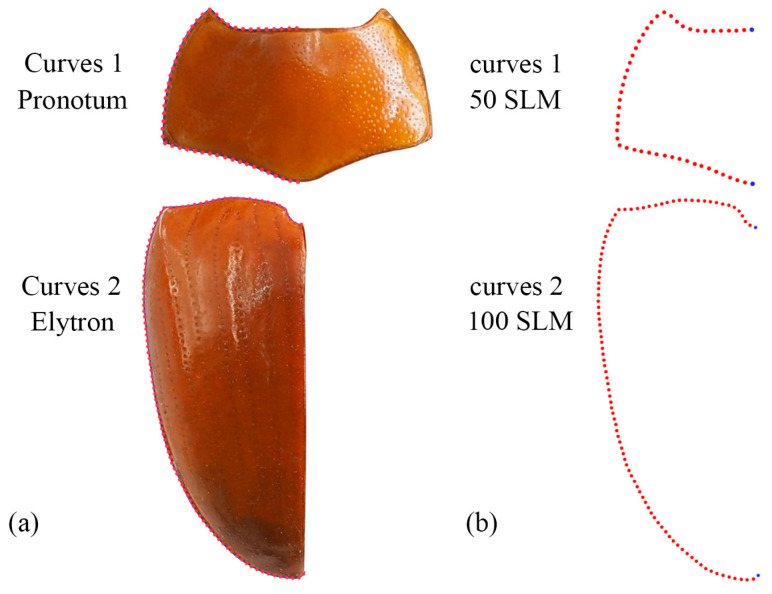
Description of the curves (**a**) and semi-landmarks (SLM) (**b**) of the pronotum and elytron used in the geometric morphometric analysis (*Neotriplax miwai*). The blue dots stand for starting and ending points.

**Figure 2:**
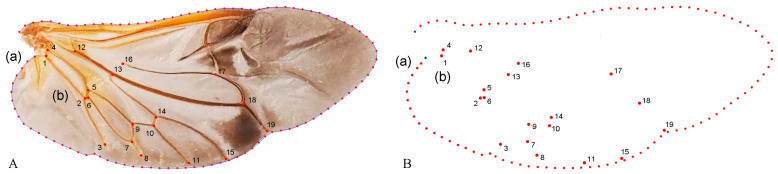
Description of the curves of the wing shape (**A**(a)) and semi-landmarks (SLM). (**B**(a)); Distribution of veins (**A**(b)) and 19 landmarks (**B**(b)) in the wing used in the geometric morphometric analysis (*Neotriplax arisana*). The blue dots stand for starting and ending points.

Geometric morphometric information was obtained from the left contour of the pronotum, elytra, and wing. The curve of the pronotum was taken from the midpoint of the anterior margin to the midpoint of the posterior margin. The curve of the elytron was taken from the boundary between the anterior margin of the elytron and the apex of the scutellar shield to the apex of the laterior margin of the elytron. The curves for the pronotum or elytron were resampled by length after 50 and 100 semi-landmarks (SLM), respectively, to ensure that their morphological information could be extracted completely (Figure 1). The curve of the wing shape was taken from the costal margin humeral angle, punctuated in a clockwise direction, and it ended at the apex of the inner margin, which was resampled by length after 100 SLM. All curves were digitized by the software TpsUtil v1.46 and Tps-Dig v2.12 [69,70,71]. For the wing vein, the intersection points and endpoints of each pulse were selected as the landmark, creating a total of 19 landmarks (Figure 2).

TpsRelw v1.74 (with the sliders file included) was used to save the aligned file [72]. The shape differences among these taxa were inferred using principal component analysis (PCA) and canonical variate analysis (CVA) in MorphoJ v1.07 [73]. MorphoJ v1.07 [73] was used to calculate the Procrustes distance and Mahalanobis distance between the morphological data of each group to evaluate the degree of difference between the average morphologies of each group.

## 3. Results

### 3.1. Phylogenetics of the Genus of Neotriplax

**Figure 3:**
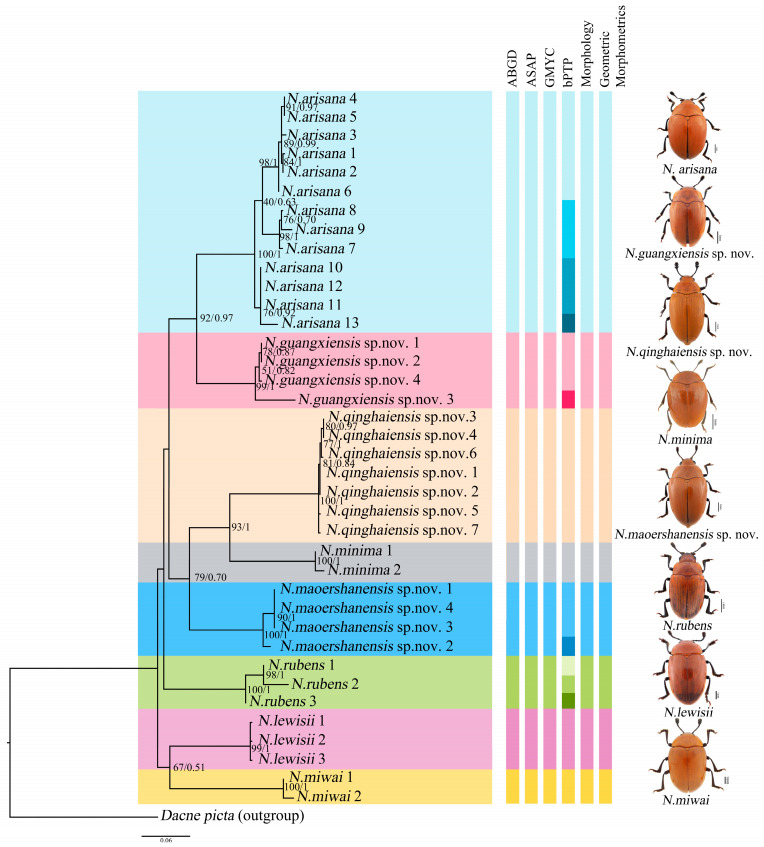
Phylogenetic relationships among the genus of *Neotriplax*. Shown here is the phylogeny inferred from the *COI* gene using ML and BI. The number on the left represents ultrafast bootstrap support (BS, %) and the number on the right represents the posterior probabilities (PP).

Phylogenetic analyses were performed on the mt DNA *COI*. A total of 39 *COI* sequences representing nine species (including the outgroup *Dacne picta*) were included in the final dataset. The *COI* alignment (585 bp; 33.4% T, 19.7% C, 30.6% A, 16.3% G) included 346 conserved sites, 239 variable sites, and 183 parsimony informative sites. The results of the phylogenetic analyses based on BI and ML reached the same topology, and almost all the nodes were highly supported (Figure 3).

The phylogenetic analyses of *Neotriplax* revealed that all groups were defined as monophyletic with high ultrafast bootstrap support (BS) and posterior probabilities (PP), and it can be seen that these species can be well divided. All the species were divided into eight clades (8 species), including five known species and three new species, *N. qinghaiensis* sp. nov., *N. maoershanensis* sp. nov. and *N. guangxiensis* sp. nov. The first clade (*N. miwai*–*N. lewisii*) was the sister to all the other species. The remaining taxa constituted three clades. *N. rubens* occurred as the sister group with the remaining other taxa. The second clade contained *N. maoershanensis* sp. nov., occurring as sister group with *N. qinghaiensis* sp. nov. and *N. minima*. The crown clade consists of *N. arisana* and *N. guangxiensis* sp. nov.

### 3.2. Genetic Distances and Species Delimitation

The Kimura-2-parameter (K_2_P) distances between different species of *Neotriplax* ranged from 0.1256 to 0.2084, with the smallest distance being between *N. guangxiensis* sp. nov. and *N. arisana* and the largest distance being between *N. qinghaiensis* sp. nov. and *N. miwai*. All K_2_P distances between the identified species were greater than 0.1256 (Table 1).

The results showed that the average inter-GD ranged from 0.0034 (*N. lewisii*) to 0.0333 (*N. arisana*), with no overlap with the genetic distances of interspecies, and the difference was obvious (Table 1).

The result of the ABGD was generated by DNA barcode, and the initial partition and recursive partition were obtained through K_2_P model analysis, as shown in Figure S1. Refer to Figure 3 for species division results. It was divided into eight molecular operational taxonomic units (MOTUs), which was consistent with morphology and phylogeny. The result of ASAP showed that species were divided into eight MOTUs based on the ASAP-score (Treshold dist. = 0.081), which was consistent with those obtained by the ABGD method. Both the results based on distance distribution supported the classification status of these new and known reddish-brown species in *Neotriplax*. The GMYC analysis based on DNA barcoding yielded eight ML clusters (without outgroup), a confidence interval of 3–10, supporting eight morphospecies, and the results were consistent with the topology of ML and BI (Figure S2). For the analysis of bPTP, these specimens formed 15 MOTUs, and *N. guangxiensis* sp. nov. and *N. maoershanensis* sp. nov. formed two MOTUs, respectively. *N. rubens* formed three MOTUs and *N. arisana* formed four MOTUs (Figure 3). However, all of these showed an indistinct boundary in ABGD, ASAP and GMYC.

Therefore, significant genetic differentiation had occurred between the species of *Neotriplax*, and DNA barcoding could better distinguish different species in the genus.

### 3.3. Geometric Morphometrics

#### 3.3.1. Geometric Morphometrics Analyses of Pronotum across Groups

**Figure 4:**
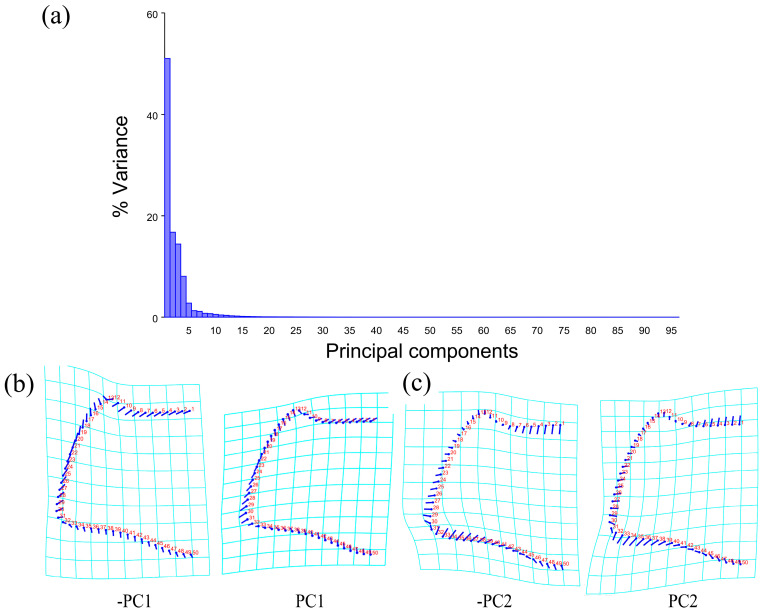
Shape variation in pronotum in *Neotriplax*. (**a**) The proportion of the total variation explained by each principal component based on the contour of the pronotum. (**b**)Variation in the pronotum along PC1. (**c**) Variation in the pronotum along PC2.

**Figure 5:**
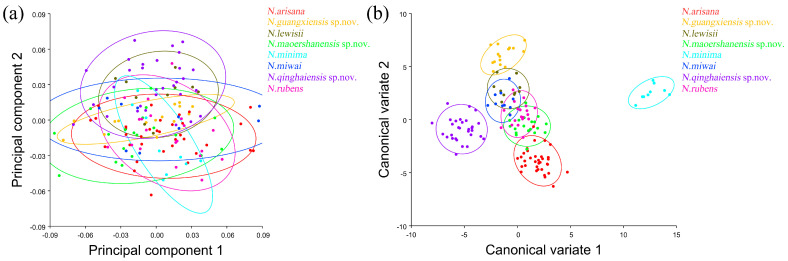
The pronotum morphological variations in *Neotriplax* based on PCA (**a**) and CVA (**b**). The 90% equal frequency ellipses containing approximately 90% of the data points.

The PCA of the pronotum showed that the first two components accounted for 67.789% of the total variation in *Neotriplax* (the first principal component, PC1: 51.036%; the second principal component, PC2: 16.753%) (Figure 4a). The overall shape change trend of the pronotum can be obtained from the shape variation map obtained by the PCA (Figure 4b,c). PC1 changed in the positive direction. The anterior border and posterior angle had a trend of extending outwards, while the anterior angle and posterior border had a trend of shrinking inwards. Overall, the aspect ratio of the pronotum increased; PC2 changed in the positive direction. The lateral and posterior border had a trend of shrinking inwards, while the anterior border and the posterior angle had a trend of extending outwards. Overall, the aspect ratio of the pronotum decreased. The results showed that all taxa clustered together with less morphological variation (Figure 5a).

The vector space constructed by the two canonical variates (CV1, CV2) with the largest proportion was employed to test the canonical variate of the pronotum contour shape variation (Figure 5b). *N. qinghaiensis* sp. nov. and *N. minima* were clearly separated from other taxa without overlapping. Both *N. arisana* and *N. guangxiensis* sp. nov. showed a minor overlap with the remaining taxa but can still be distinguished with others. However, there are many overlaps between *N. rubens*, *N. lewisii*, *N. maoershanensis* sp. nov., and *N. miwai* which cannot be distinguished.

In terms of statistical test parameters (Table S3), the Mahalanobis distance of *N. maoershanensis* sp. nov. and *N. miwai* was the smallest, *N. miwai* and *N. minima* was the largest, at 6.1333 and 18.2966, respectively, and the difference was extremely significant (*p* < 0.01). *N. qinghaiensis* sp. nov. and *N. lewisii* had the smallest Procrustes distance and *N. minima* had the largest Procrustes distance, at 0.0206 and 0.0756, respectively, and the difference was extremely significant (*p* < 0.01).

#### 3.3.2. Geometric Morphometrics Analyses of Elytron across Group

**Figure 6:**
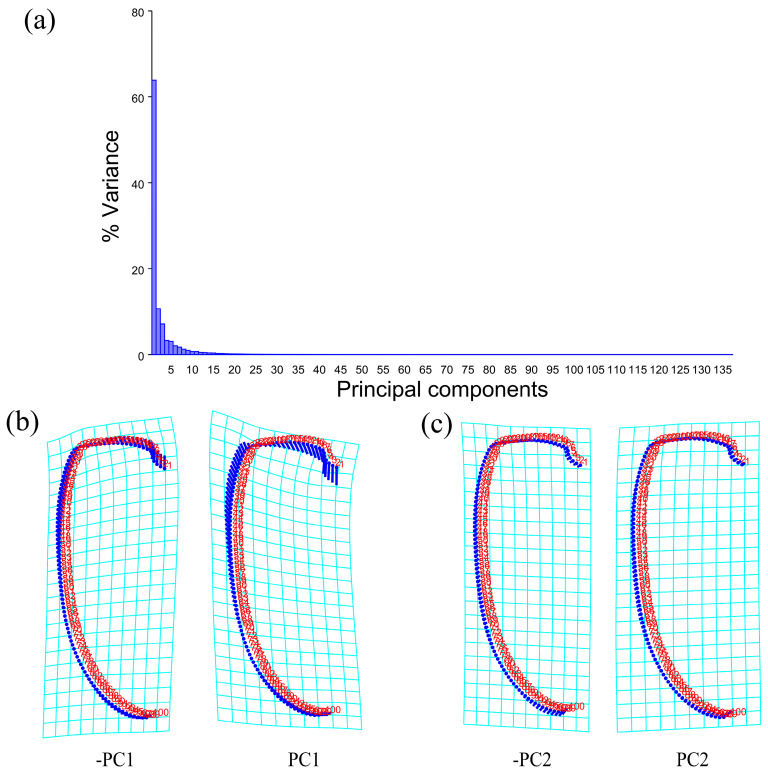
Shape variation trend of elytron in *Neotriplax*. (**a**) The proportion of the total variation explained by each principal component based on the contour of the elytron. (**b**) Variation in the elytron along PC1. (**c**) Variation in the elytron along PC2.

**Figure 7:**
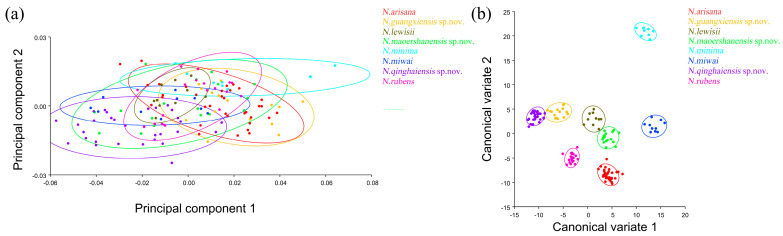
The elytron morphological variations in *Neotriplax* based on PCA (**a**) and CVA (**b**). The 90% equal frequency ellipses containing approximately 90% of the data points.

The PCA of elytron showed that the first two components accounted for 74.530% of the total shape variation in the elytron of *Neotriplax* (PC1:63.858%; PC2:10.671%) (Figure 6a). The change trend of the contours of the elytron can be obtained from the shape variation map obtained by the PCA of the elytron (Figure 6b,c). PC1 changes in the positive direction. The scutellum, anterior, and posterior border of the elytron had a trend of shrinking inwards, while the lateral border had a trend of extending outwards. The shape change trend was not large, and the aspect ratio decreased; PC2 changed in the positive direction. The scutellum and lateral border of the elytron had a trend of extending outwards, while the anterior and posterior border had a trend of shrinking inwards. The shape variation was not obvious. The results showed that all taxa clustered together with less morphological variation (Figure 7a). Based on the canonical variates of CV1 and CV2, all species were clearly separated from other taxa without overlapping. (Figure 7b).

In terms of statistical test parameters (Table S4), the Mahalanobis distance between *N. qinghaiensis* sp. nov. and *N. guangxiensis* sp. nov. was the smallest, and the Mahalanobis distance between *N. minima* and *N. rubens* was the largest, at 13.4801 and 31.9947, respectively, and the difference was extremely significant (*p* < 0.01). *N. rubens* and *N. maoershanensis* sp. nov. had the smallest Procrustes distance, and *N. qinghaiensis* sp. nov. and *N. minima* had the largest Procrustes distance, at 0.0084 and 0.0561, respectively, and the difference was extremely significant (*p* < 0.01).

#### 3.3.3. Geometric Morphometrics Analyses of Wing Shape and Wing Vein across Group

**Figure 8:**
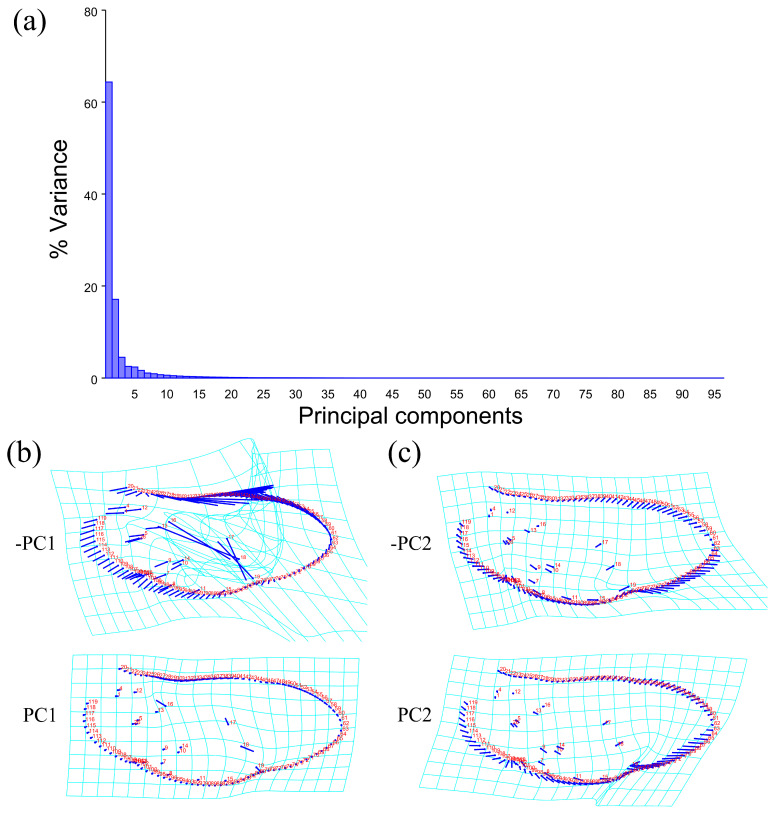
Shape variation trend of hind wing shape and wing vein in *Neotriplax*. (**a**) The proportion of the total variation explained by each principal component based on contour of wing shape and wing vein. (**b**)Variation in the wing shape and wing vein along PC1. (**c**) Variation in the wing shape and wing vein along PC2.

The PCA of the wing shape and wing vein showed that the first two components accounted for 81.432% of the total shape variation in the elytron of *Neotriplax* (PC1: 64.364%; PC2: 17.068%) (Figure 8a). The change trend of the contours of the wing shape and the landmarks of the wing vein (Figure 8b,c) can be obtained from the shape variation map obtained by the PCA. PC1 changed in the positive direction. Points 1–15, 18, 19 tended to move in the opposite direction to the base of the wing, while points 16, 17 tended to move towards the base of the wing. The costal margin humeral angle and the outer margin of the hind wing had a trend of extending outwards, and the costal margin half and inner margin had a trend of shrinking inwards.

**Figure 9:**
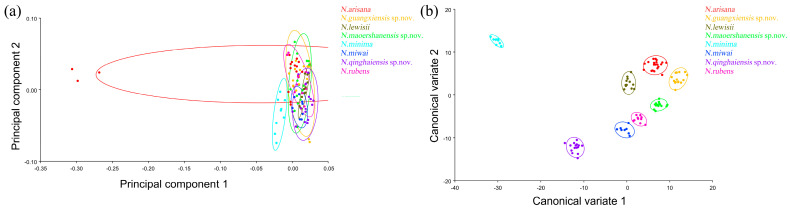
The wing shape and wing vein morphological variations in *Neotriplax* based on PCA (**a**) and CVA (**b**). The 90% equal frequency ellipses containing approximately 90% of the data points.

PC2 changed in the positive direction. Points 1–19 tended to move in the opposite direction to the base of the wing. The outer margin had a trend of shrinking inwards, while the costal and inner margin, as well as the apical angle, tended to expand outwards. The results showed that all taxa clustered together with less morphological variation (Figure 9a).

Based on the canonical variate of CV1 and CV2, all species were clearly separated from other taxa without overlapping (Figure 9b).

In terms of statistical test parameters (Table S5), the Mahalanobis distance between *N. maoershanensis* sp. nov. and *N. rubens* was the smallest, and the Mahalanobis distance between *N. minima* and *N. guangxiensis* sp. nov. was the largest, at 14.8569 and 43.7562, respectively, and the difference was extremely significant (*p* < 0.01). *N. guangxiensis* sp. nov. and *N. maoershanensis* sp. nov. had the smallest Procrustes distance, and *N. miwai* and *N.arisana* had the largest Procrustes distance, at 0.0189 and 0.0815, respectively.

Taking into account all three results, all eight morphospecies have a large degree of shape variation and can be effectively differentiated.

### 3.4. Taxonomy of Neotriplax

Through morphological research, geometric morphometric analysis, genetic distance, species delimitation, and phylogenetic construction, the taxonomic status of reddish-brown *Neotriplax* were clarified, and *N. guangxiensis* sp. nov., *N. maoershanensis* sp. nov. and *N. qinghaiensis* sp. nov. were determined as new species. Our taxonomic study confirmed the accuracy of diagnostic characteristics that were useful on a species level; for similar species of this genus, valid and objective diagnostic characteristics include the presence or absence of coxal lines, the shape and aspect ratio of the pronotum, the shape of the mentum and submentum, the shape of the prosternal process, and the shape of the scutellar shield.

Genus *Neotriplax* Lewis, 1887

Type species: *Neotriplax atrata* Lewis, 1887.

Key to Species of the Genus *Neotriplax*

1. Body coloration mostly or completely black…2.
  - Body coloration reddish-brown…5.
2. Body coloration uniformly black…3.
  - Body coloration not monochrome black…4.
3. Body with the sides less rounded; maxillary terminal palpomere transverse subtriangular, with the sides distinctly rounded…*N. atrata*.
  - Body with the sides a little more rounded; maxillary terminal palpomere triangular, with the inner margin evenly but very slightly arched and the apical angle rather acute…*N. delkskampi*.
4. General color black, antennae deeply reddish-brown, labial yellowish-brown; elytron humerus with red patches…*N. biplagiata*.
  - The laterial margins of elytra yellow, abdomen deeply reddish-brown…*N. pallidicincta*.
5. Pronotum 2.5 times as wide as long…*N. rubens*.
  - Pronotum width/length ≤ 2.0…6.
6. All coxal lines present…7.
  - Prosternal, postmeso- and postmetacoxal lines incomplete or absent…11.
7. Posterior border of pronotum strongly narrowed backwards, with a narrow lobe in middle…*N. minima*.
  - Posterior border of pronotum slightly narrowed backwards, with a broad lobe in middle…8.
8. Mentum rolled up on both sides, submentum broader; lateral margins of pronotum strongly curve from base 1/2 to the top…*N. guangxiensis* sp. nov.
  - Mentum flat on both sides, submentum narrower; lateral margins of pronotum straight…9.
9. Mentum with triangular in bump, without middle area depressed…*N. maoershanensis* sp. nov.
  - Mentum with pentagon in bump, and middle area triangularly depressed…10.
10. Scutellar shield broad; anterior margin of prosternal process slightly straight…*N. miwai*
  - Scutellar shield narrow; anterior margin of prosternal process strongly emarginated…*N. arisana*.
11. All coxal lines absent…*N. qinghaiensis* sp. nov.
  - Only postmesocoxal lines absent…*N. lewisii*.

**Figure 10:**
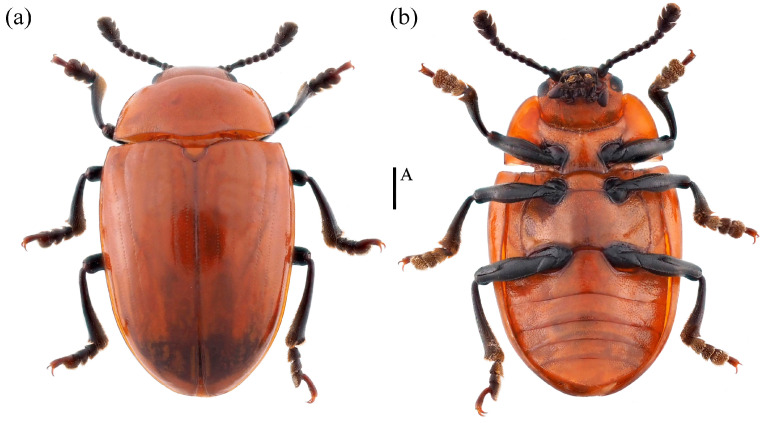
Dorsal and ventral habitus of *Neotriplax lewisii* Crotch, 1873. (**a**) Dorsal view; (**b**) ventral view, scales: 1 mm (A).

*Neotriplax lewisii* Crotch, 1873 (Figure 10) New Record in China

*Neotriplax lewisii* Crotch, 1873: 189.

Material examined. 1♂1♀: China, Guangxi Province, Ziyuan City, Mt. Yinzulao Nature Reserve, 26.040908 E, 110.675830 N, 26 August 2013, Yiping Niu leg; 2♀: China, Chongqing City, Chengkou County, Libanping Village, 31.734000 E, 108.942000 N, 17 June 2017, Bin Chen leg; 1♀: China, Guangxi Province, Huaping Nature Reserve, 25.604400 E, 109.904183 N, 28 February 2021, Panpan Li leg.

Body length: 4.2–8.1 mm; width: 2.4–3.4 mm.

Distribution: New for China (Guangxi, Chongqing); Japan, South Korea.

Comparative notes. All specimens are similar to the type specimen, but considerable interspecific variability of *N. lewisii* was observed in the body size (Figure 10).

**Figure 11:**
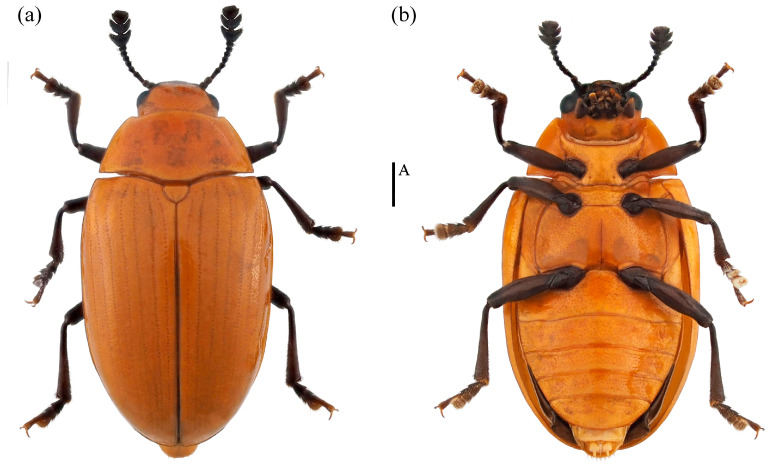
Dorsal and ventral habitus of *Neotriplax qinghaiensis* Liu and Li, sp. nov. (**a**) Dorsal view; (**b**) ventral view, scales: 1 mm (A).

*Neotriplax qinghaiensis* sp. nov. (Figure 11)

Material examined. *Holotype*. ♀: China, Qinghai Province, Menyuan County, Mt. Qilian National Park,37.130644 E, 102.435669 N, 28 July 2021, Xinglong Bai leg (deposited in MHBU). *Paratypes*, 1♂1♀: China, Qinghai Province, Menyuan County, Mt. Qilian National Park, 37.085211 E, 102.370556 N, 24 July 2021, Xinglong Bai leg (deposited in MHBU); 2♀: China, Qinghai Province, Menyuan County, Mt. Qilian National Park, 37.131794 E, 102.399081 N, 24 July 2021, Xinglong Bai leg (deposited in MHBU); 1♂1♀: China, Qinghai Province, Maixiu National Park, 35.160360 E, 101.560260 N, 18 August 2019, Xinglong Bai leg (deposited in HEBAU).

Description. Body elongate-oval, distinctly convex dorsally, smooth and slightly glossy. General color reddish-brown, abdomen bright yellow, clypeus, mouthparts, eyes, antennae and legs black.

**Figure 12:**
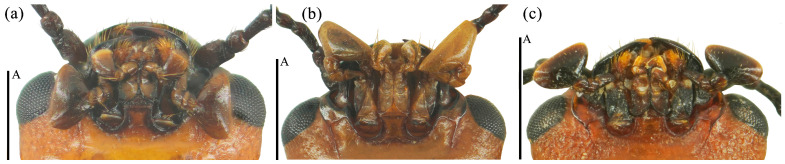
The mouthparts of *Neotriplax qinghaiensis* sp. nov. (**a**); *Neotriplax maoershanensis* sp. nov. (**b**); *Neotriplax guangxiensis* sp. nov. (**c**). Scales: 0.5 mm (A).

Head large with coarse and sparse punctures. Clypeus emarginated at the anterior border. Frontoclypeal suture completed. Compound eye small, prominent, interocular distance is equal to 4.3× width of eye radius. Antennae long and robust, with short golden setae, antennomere 1 robust; antennomere 2 small, nearly spherical; antennomere 3 longer than antennomere 4; antennomeres 5–7 nearly equal; antennomeres club transverse and compact, antennomere 9 bowl-shaped; antennomere 10 crescent, antennomere 11 near pentagon and narrower than the former two segments, with depression in middle; relative lengths of antennomeres 2–11: 1.5: 2.6: 1.8: 1.7: 1.7: 1.75: 1.5: 3.5: 3.4: 3.5. Maxillary terminal palpomere axe-shape, nearly 1.74× as wide as long. Labial terminal palpomere cylindrical. Mentum triangular, both sides with marginal border, middle area depressed. Submentum nearly narrow, smooth and without punctures (Figure 12a).

Pronotum transverse, nearly 1.96x as wide as long, convex dorsally, finely punctured. Anterior margin with narrow and complete marginal border, lateral margins with distinctly marginal border. Anterior angles blunt tip, posterior angles almost rectangular. Prosternum with coarse punctures in middle, anterior margin slightly emarginated with narrow marginal border, the middle of base slightly emarginated. Prosternal lines absent. Scutellar shield large, nearly semicircle, arc at the apex, without punctures. Elytra long, 1.5x as long as wide, more convex dorsally than the pronotum, widest at the base 1/3.

Mesoventrite transverse, smooth and with punctures. Metaventrite coarsely punctured at sides and indistinctly in middle. Postmesocoxal lines absent. Abdomen with dense and fine punctures. Postmetacoxal lines absent.

Legs long and fine, femora slightly emarginated at the inside of apex, tibiae slightly transverse at the apex, but not triangular, the 1–3 segments of tarsus gradually widening to apex.

**Figure 13:**
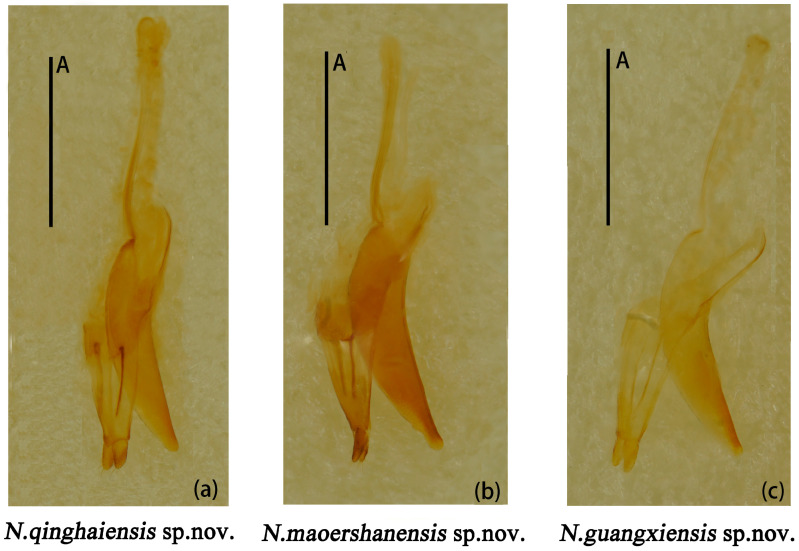
*Neotriplax qinghaiensis* sp. nov. (**a**); *Neotriplax maoershanensis* sp. nov. (**b**); *Neotriplax guangxiensis* sp. nov. (**c**). Aedeagus in lateral views, scales: 1 mm (A).

Male genitalia median lobe curved, apex narrow and about 1.1× as long as median strut (Figure 13a).

**Figure 14:**
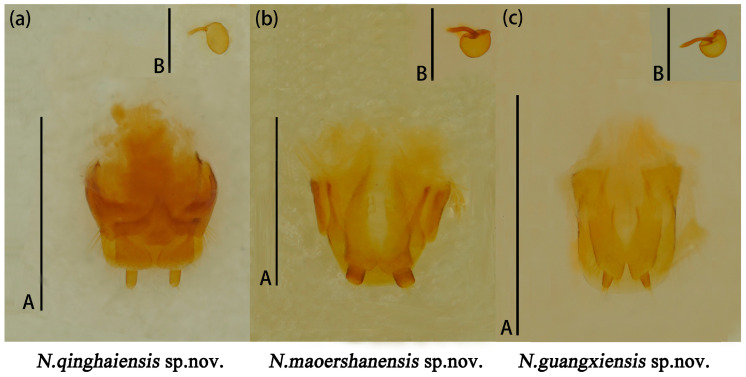
*Neotriplax qinghaiensis* sp. nov. (**a**); *Neotriplax maoershanensis* sp. nov. (**b**); *Neotriplax guangxiensis* sp. nov. (**c**). Female genitalia and female spermatheca. Scales: 1 mm (A); scales: 0.5 mm (B).

Female genitalia small, proctigeral lobe small, coxite rather robust, stylus small, nearly cylindrical. Spermatheca nearly spherical (Figure 14a).

Body length: 4.2–6.7 mm; width: 2.4–3.4 mm.

Remarks. The body shape and color of this species are similar to *N*. *lewisii*. This new species can be identified by its slightly narrower body, with prosternal lines, postmesocoxal lines, and postmetacoxal lines absent. *N*. *lewisii* has a broader body, with only postmesocoxal lines absent, while prosternal lines and postmetacoxal lines are present.

Distribution: China (Qinghai).

Etymology. The specific name is derived from the type locality, Qinghai Province, China.

**Figure 15:**
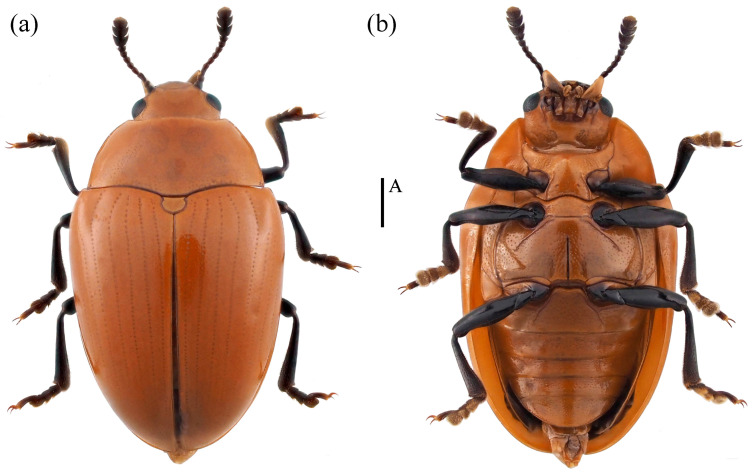
Dorsal and ventral habitus of *Neotriplax maoershanensis* Liu and Li, sp. nov. (**a**) Dorsal view; (**b**) ventral view, scales: 1 mm (A).

*Neotriplax maoershanensis* sp. nov. (Figure 15)

Material examined. *Holotype*. ♀: China, Guangxi Province, Liuzhou City, Mt. Maoer, 24.379590 E, 110.433958 N, 2 June 2011, Qing Zhang and Hailing Wang leg (deposited in MHBU). *Paratypes*, 1♀: China, Guangxi Province, Huaping Nature Reserve, Cujiang Station, 25.604500 E, 109.903700 N, 5 May 2020, Panpan Li leg (deposited in IZAS); 1♂: China, Guangxi Province, Huaping Nature Reserve, Cujiang Station, 25.603133 E, 109.907600 N, 14 April 2020, Panpan Li leg (deposited in IZAS); 1♂: China, Guangxi Province, Huaping Nature Reserve, Anjiangping Station, 25.601389 E, 110.049444 N, 1 July 2020, Panpan Li leg (deposited in IZAS).

Description. Body elongate-oval, convex dorsally, smooth and shining. General color reddish-brown, abdomen dark reddish-brown, eyes, antennae, and legs black.

Head small, the anterior border of clypeus emarginated. Frontoclypeal suture completed. Compound eye small, interocular distance is equal to 3.9× width of eye radius. Antennae short and slender, antennomere 1 short but robust; antennomere 2 small, antennomere 3 long, equal in length with antennomere 4 and 5 combined, antennomere 4 nearly spheric, antennomere 6 and 7 nearly equal; antennomere 8 slightly shorter than antennomere 7; antennomeres club compact, antennomere 10 more compact with antennomere 11 than 9; antennomere 9 bowl-shaped; antennomere 10 crescent-shaped; antennomere 11 nearly rounded, with depression in middle; relative lengths of antennomeres 2–11: 3.9: 6.9: 3.6: 3.4: 3.1: 2.7: 2.9: 6.1: 5.1: 6.0. Maxillary terminal palpomere triangular, 1.9× as wide as long. Labial palpomere short, with the terminal segment cylindrical and truncated at the apex. Mentum with middle area triangular depressed, submentum narrow (Figure 12b).

Pronotum nearly trapezoidal, 2× as wide as long, with fine and uniform punctures and denser than head. Anterior margin shallowly bisinuate, lateral margins straight in basal half, converges forward in middle, with distinct and complete marginal border, basal margin with complete marginal border, weakly bisinuate. Anterior angles acute, protruded and blunt, posterior angles blunt, each with one pore. Prosternum with fine and sparse punctures, anterior border produced to short point in middle, with distinct marginal border. Prosternal process with depression at the apical emargination and with one pore at each side. Prosternal lines short, converge slightly forward. Scutellar shield nearly semicircle, round posteriorly. Elytra long and wide, 1.3× as wide as long, with eight striae, internals extremely fine punctures, widest at the base 1/6.

Mesoventrite transverse, each side with one shallow depression, with coarse punctures. Metaventrite with coarse and shallow punctures. Postmesocoxal lines extending to basal 1/2 of metaventrite. Abdomen with dense punctures, postmetacoxal lines long, and extending to basal 3/4 of ventrite 1.

Legs short and slender, femora robust, tibiae slightly transverse at the apex, tarsus 1–3 gradually widening.

Male genitalia (Figure 13b) median lobe weakly curved, gradually narrow from base to apex; median strut straight, 0.9× as long as median lobe.

Female genitalia (Figure 14b) small, stylus nearly broadly cylindrical. Female spermatheca nearly spherical.

Body length: 6.8–7.5 mm; width: 3.7–4.5 mm.

Remarks. This new species is similar to *N*. *arisana* in terms of body color and shape. It is distinguished by its narrow triangular mentum, narrower submentum, and yellow maxillary and labial palpomeres. In contrast, *N*. *arisana* has a pentagonal mentum, broader submentum, and black maxillary and labial palpomeres.

Distribution: China (Guangxi).

Etymology. The specific name is derived from the type locality.

**Figure 16:**
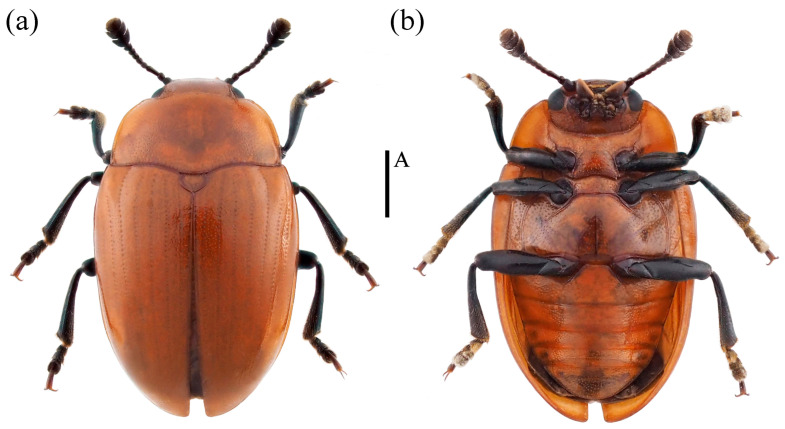
Dorsal and ventral habitus of *Neotriplax guangxiensis* Liu and Li, sp. nov. (**a**) Dorsal view; (**b**) ventral view, scales: 1 mm (A).

*Neotriplax guangxiensis* sp. nov. (Figure 16)

Material examined. *Holotype*. ♂: China, Guangxi Province, Guilin City, Guanyang County, Yezhudian Village, 25.230761 E, 110.733406 N, 6 May 2021, Weicheng Lu leg (deposited in HEBAU). *Paratypes*, 1♂1♀: China, Guangxi Province, Huaping Nature Reserve, Anjiangping Station, 25.765833 E, 109.942500 N, 1 July 2020, Panpan Li leg (deposited in IZAS); 1♂: China, Guangxi Province, Huaping Nature Reserve, Cujiang Station, 25.604500 E, 109.903700 N, 5 October 2020, Panpan Li leg (deposited in IZAS).

Description. Body oval, convex dorsally, smooth and glossy. General color reddish-brown, clypeus, antennae, eyes, mouthparts and legs black.

Head large, with coarse and dense punctures. Clypeus emarginated in middle of anterior border. Frontoclypeal suture completed. Compound eyes small, finely faceted. Antennae short and robust, with golden setae. Antennomere 1 robust; antennomere 2 small, nearly round; antennomere 3 longer than others, nearly equal to 4 and 5 combined; antennomere club transverse, antennomere 9 deep bowl-shape; antennomere 10 crescent-shaped; antennomere 11 oval, with depression in middle, slightly smaller than 10; antennomere 10 more compact with antennomere 11 than 9. Relative lengths of antennomere 2–11: 2.1: 3.5: 1.8: 1.7: 1.5: 2.0: 1.6: 1.5: 3.3: 3.1: 3.4. Maxillary terminal palpomere semicircle, 1.7x as wide as long. Mentum upturned on both sides, triangular in bump, with middle area depressed, submentum broad (Figure 12c).

Pronotum slightly trapezoidal, convex dorsally, 1.8x as wide as long, with fine and densely punctures. Anterior margin with narrow and completed marginal border, shallowly forward projection in middle; lateral margins broadly rounded, converges forward, with distinct marginal border. Basal border weakly sinuate. Anterior angles acute, blunt tip, posterior angles blunt. Prosternum impunctate laterally, with sparse punctures medially, prosternal process with one indistinctly pore at each side. Prosternal lines long, converge forward. Scutellar shield large, nearly heart-shaped, angulate posteriorly, with fine punctures. Elytra long, 1.3× as long as wide, with eight striae, internals extremely fine punctures.

Mesoventrite transverse, with coarse punctures. Metaventrite with dense and coarse punctures on each side of base and without punctures in middle. Postmesocoxal lines long, extending to basal 1/2 of metaventrite. Abdomen with dense and fine punctures. Postmetacoxal lines extending to basal 1/2 of ventrite 1.

Legs long, femora robust, tibiae slightly transverse at apex. Tarsus 1–3 gradually widening.

Male genitalia (Figure 13c) median lobe curved, nearly equal in length to median lobe.

Female genitalia (Figure 14c) stylus narrow from base to end; female spermatheca nearly oval.

Body length: 5.0–5.2 mm; width: 2.6–3.1 mm.

Remarks. This species is similar to *N. arisana* in terms of body color and shape. It is distinguished by the lateral margin of the pronotum and is strongly curved from the base 1/2 to top. The anterior angle is protruded and blunt; the mentum is upturned on both sides, triangular in bump, and the submentum is broad. In contrast to the new species, *N. arisana* has its lateral margin of the pronotum straight. The mentum is flat on both sides, pentagon in bump, submentum width centering.

Distribution: China (Guangxi).

Etymology. The specific name is derived from the type locality, Guangxi Province, China.

## 4. Discussion

An integrative taxonomy analysis was firstly provided by comprehensively utilizing morphology, molecular phylogeny, and geometric morphometrics, which supported the relationship between all known and new reddish-brown species by integrative taxonomy. Including the three new species described here, *Neotriplax* now comprise 12 species. The results have increased the biodiversity of this genus within China and worldwide.

Currently, the application of integrative taxonomy in the insect species delimitation has achieved remarkable results, such as in the species delimitation of *Bactrocera dorsalis*, *Machilis*, *Encyrtus sasakii*, and other insects [74,75,76]. This comprehensive taxonomy approach has been proven to provide a reference for distinguishing closely related species, which deepen the understanding and knowledge of the boundaries between species.

### 4.1. Phylogeny of Neotriplax

This study provides the first insight into the phylogenetic relationships within the genus *Neotriplax*. In the phylogenetic tree constructed using the ML and BI methods, all these species are divided into eight clades, and all of them are monophyletic. Our study supports the current taxonomic status of reddish-brown species in *Neotriplax* in terms of phylogeny, which is consistent with the results of the morphological research [7,8,13]. Among them, *N. arisana* and *N. guangxiensis* sp. nov. have a close phylogenetic relationship, and they also resemble each other morphologically.

DNA barcoding has been proven to be a powerful method for rapid species identification [77], and our study further validates its application in *Neotriplax*. These similar species showed an obvious barcoding gap. For the first time, we analyzed the inter- and intraspecific genetic distance threshold of *Neotriplax*, considered as a crucial basis for species delimitated [78,79,80,81], revealing that the minimum inter-GD was significantly larger than the maximum intra-GD and the threshold of genetic distance was set at 0.1256 can be used to distinguish between species.

### 4.2. Molecular Species Delimitation

The four approaches of molecular species delimitation showed two different results. The methods ABGD, ASAP, and GMYC unanimously recognized eight MOTUs, aligning with the morphospecies. However, the bPTP approach revealed a discrepancy, identifying a total of 15 MOTUs. Obviously, our results indicate that the bPTP method yields more putative species than suggested by other methods and morphology-based classification. This method has previously shown a tendency to over-split species [82]. The bPTP method relies on ML trees; species delimited with a well-defined morphology may be more accurate than with GMYC [28,83]. Therefore, a comprehensive consideration using multi-gene and multi-method approaches is needed. These results proved that morphological and molecular species definition can be effectively combined to improve the accuracy of species classification and provide data support for molecular species definition and integrated taxonomic research on this genus.

### 4.3. Geometric Morphometrics

Geometric morphometrics can be used for the rapid identification of insects at lower taxonomic levels [84,85]. First, the pronotum and elytron, which have rich morphological diversity and powerful functions, are considered continuous characteristics [86,87,88]. The geometric morphometrics of the pronotum and elytra have been well applied in the identification of related genera and species of Coleoptera, the classification of geographical populations, and the study of biological evolution [88]. However, in *Neotriplax*, the continuous characteristics of the pronotum did not distinguish all similar species, indicating that the shape variation in the pronotum is not applicable in the geometric morphological analysis of *Neotriplax*. This finding is consistent with the previous results, suggesting the shape variation in the pronotum is not reliable for the classification of lower-level taxa [89].

Subsequently, this study further selected the hind wings for geometric morphological analysis. Hind wing characteristics, such as wing shape and wing veins, are important in taxonomic and phylogenetic analyses [90,91,92]. Combining the shape variations in the wing shape and wing veins, we can distinguish closely related species, which also supports the accuracy of the status of these species. This also shows that the shape variations in the hind wing can be used as a taxonomic basis to distinguish *Neotriplax* species, ensuring that all these species are correctly classified.

Since genitalia can reflect information about insect mating behavior, evolutionary history, and other aspects, they can also be used as an auxiliary basis in some specific cases [93,94]. However, due to the limitation of the number of unisexual samples, it has increased the difficulty of comparative morphological studies. In future work, we need to further collect samples and conduct comparative morphological analysis of genitalia.

Geometric morphometrics provides a valuable reference for the classification of the genus. Moreover, it significantly improves the accuracy of identifying closely related and morphologically similar species. By comprehensively analyzing quantitative morphological and molecular data, more reliable classification results can be obtained through integrative taxonomic studies.

### 4.4. Integrated Taxonomy

In the process of species identification, adopting different classification methods often leads to differences in classification results. This is primarily due to the different perspectives and focuses of different classification methods [95].

Morphology is the foundation of species diversity research, providing support for the discovery, description, and identification of species [96]. The characteristics of geometric morphometrics lie in its ability to eliminate factors such as the size, color, and so on, focusing only on the difference of shape variations. Moreover, it can quantitatively analyze continuous traits, which offers great advantages in its application in the study of species delimitation among closely related species [46,89]. However, the phenotype of species is influenced by both genetic and environmental factors, which increases the uncertainty in the identification [95,97].

Molecular analysis not only provides strong technical support for species delimitation but also offer a new technical reference for the discovery of new species and cryptic species [98]. However, while molecular techniques exhibit unique advantages in species delimitation, primarily focusing on revealing genetic differences within and between species, there are still potential limitations, and relying solely on molecular data for species delimitation may lead to underestimation or overestimation of species diversity [81].

Integrated taxonomy aims to provide more comprehensive and accurate species identification results by comprehensively utilizing multiple taxonomy methods, providing a more reliable foundation for biological research [99]. By integrating multiple taxonomy evidence and techniques [100,101,102], species delimitation can be performed more accurately, which can provide a more reliable basis for the protection and utilization of biodiversity.

## Figures and Tables

**Table 1:** The Kimura 2-parameter (K_2_P) distances matrix between the taxa of *Neotriplax* based on the *COI* gene (diagonal is the intra-GD).

Species	1	2	3	4	5	6	7	8
*N. arisana*	0.0333							
*N. guangxiensis* sp. nov.	0.1256 *	0.0246						
*N. lewisii*	0.1516	0.1433	0.0034					
*N. maoershanensis* sp. nov.	0.1520	0.1485	0.1516	0.0121				
*N. minima*	0.1703	0.1685	0.1762	0.1637	0.0103			
*N. miwai*	0.1656	0.1702	0.1780	0.1711	0.1967	0.0121		
*N. qinghaiensis* sp. nov.	0.1810	0.1756	0.1756	0.1663	0.1609	0.2084 **	0.0041	
*N. rubens*	0.1496	0.1419	0.1536	0.1767	0.1579	0.1925	0.1807	0.0328

Note: * represents the lowest genetic distance, ** represents the highest genetic distance.

## Data Availability

All sequences were deposited in the GenBank of NCBI at https://www.ncbi.nlm.nih.gov (accessed on 5 Mar 2024) (see Table S1 for details).

## Supplementary Materials

The following supporting information can be downloaded at: https://www.mdpi.com/article/10.3390/insects15070508/s1, Table S1. A list of specimens included in this study: Table S2. Primers used for PCR in this study. Table S3. The statistical test of *Neotriplax* based on the shape variations in the pronotal outline (above the diagonal: Procrustes distance; below the diagonal: Mahalanobis distance). Table S4. The statistical test of *Neotriplax* based on the shape variations in the elytron outline (above the diagonal: Procrustes distance; below the diagonal: Mahalanobis distance). Table S5. The statistical test of *Neotriplax* based on the shape variations in the wing shape and wing vein (above the diagonal: Procrustes distance; below the diagonal: Mahalanobis distance). Figure S1. Automatic partition results by ABGD based on *COI*. Figure S2. Results of single-threshold GMYC model for *Neotriplax* based on *COI*. Species defined by a single-threshold GMYC model (a); relationship between time and lineages, red line representing the transition time of population and species (b); relationship between time and likelihood value (c).
