# Supplementary material for: Phylogenetics, Molecular Species Delimitation and Geometric Morphometrics of All Reddish-Brown Species in the Genus Neotriplax Lewis, 1887 (Coleoptera: Erotylidae: Tritomini)
Source: Insects. 2024 Jul 6;15(7):508. doi: 10.3390/insects15070508 (PMC11277550; doi:10.3390/insects15070508)
Supplement: Supplementary file 1 [file insects-15-00508-s001.zip › TableS3-S5.pdf]

Table S3 The statistical test of *Neotriplax* based on the shape variations of pronotal outline (above the diagonal: Procrustes distance; below the diagonal: Mahalanobis distance)

	N. arisana	*N. guangxiensis* sp. nov.	*N. lewisii*	*N. maoershanensis* sp. nov.	*N. minima*	*N. miwai*	*N. qinghaiensis* sp. nov.	*N. rubens*
N. arisana		0.0351**	0.0436**	0.0397**	0.0377**	0.0253	0.0502**	0.0332**
N. guangxiensis sp. nov.	10.6903**		0.0258	0.0435**	0.0553**	0.0245	0.0379**	0.0448**
N. lewisii	9.1973**	8.0197**		0.0544**	0.0654**	0.0272	0.0206	0.0506**
N. maoershanensis sp. nov.	6.7421**	9.4069**	9.0386**		0.0504**	0.0353*	0.0635**	0.0292*
N. minima	14.0737**	16.2324**	15.7302**	15.2002**		0.052**	0.0756**	0.035*
N. miwai	8.5455**	7.8558**	8.0775**	6.1333**	16.1751**		0.0373**	0.0317
N. qinghaiensis sp. nov.	9.0755**	9.899**	9.0834**	9.7323**	18.2966**	8.328**		0.0633**
N. rubens	8.9042**	10.1157**	10.1426**	7.5173**	15.3865**	8.5536**	9.4217**	

Note: * indicates that *p* value of replacement test <0.05 (10000 repetitions); ** indicates a replacement test *p* value <0.01 (10000 repetitions)

Table S4 The statistical test of *Neotriplax* based on the shape variations of elytron outline (above the diagonal: Procrustes distance; below the diagonal: Mahalanobis distance)

	N. arisana	*N. guangxiensis* sp. nov.	*N. lewisii*	*N. maoershanensis* sp. nov.	*N. minima*	*N. miwai*	*N. qinghaiensis* sp. nov.	*N. rubens*
N. arisana		0.0123*	0.0238**	0.0180**	0.0219**	0.0296**	0.0429**	0.0136**
N. guangxiensis sp. nov.	19.0644**		0.0293**	0.0257**	0.0170	0.0364**	0.0484**	0.0200**
N. lewisii	18.9277**	16.8265**		0.0124	0.0345**	0.0118	0.0245**	0.0121*
N. maoershanensis sp. nov.	17.3604**	19.4822**	21.4792**		0.0327**	0.0138	0.0297**	0.0084
N. minima	30.9421**	27.1410**	27.8156**	28.5219**		0.0422**	0.0561**	0.0265**
N. miwai	20.3243**	25.2181**	22.0482**	20.3099**	25.4747**		0.0184*	0.0184**
N. qinghaiensis sp. nov.	20.1531**	13.4801**	19.7844**	20.0861**	29.7974**	26.6318**		0.0331**
N. rubens	16.5195**	17.0224**	21.2871**	19.3402**	31.9947**	21.8970**	17.1021**	

Note: * indicates that *p* value of replacement test <0.05 (10000 repetitions); ** indicates a replacement test *p* value <0.01 (10000 repetitions)

Table S5 The statistical test of *Neotriplax* based on the shape variations of wing shape and wing vein (above the diagonal: Procrustes distance; below the diagonal: Mahalanobis distance)

	N. arisana	*N. guangxiensis* sp. nov.	*N. lewisii*	*N. maoershanensis* sp. nov.	*N. minima*	*N. miwai*	*N. qinghaiensis* sp. nov.	*N. rubens*
N. arisana		0.0600*	0.0675*	0.0573	0.0696*	0.0815*	0.0778**	0.0561*
N. guangxiensis sp. nov.	17.4943**		0.0329**	0.0189	0.0737**	0.0679**	0.0530**	0.0312*
N. lewisii	25.6584**	23.8246**		0.0304**	0.0608**	0.0524**	0.0371**	0.0287**
N. maoershanensis sp. nov.	16.3947**	14.8863**	23.9925**		0.0691**	0.0605**	0.0456**	0.0223
N. minima	39.4027**	43.7562**	38.3529**	41.1742**		0.0482**	0.0494**	0.0654**
N. miwai	24.3023**	22.1217**	26.0372**	18.8946**	38.0822**		0.0371**	0.0529**
N. qinghaiensis sp. nov.	27.6847**	30.9486**	28.5597**	24.6170**	32.3159**	21.4027**		0.0403**
N. rubens	19.4426**	18.9032**	19.5768**	14.8569**	39.1557**	17.5775**	20.6382**	

Note: * indicates that *p* value of replacement test <0.05 (10000 repetitions); ** indicates a replacement test *p* value <0.01 (10000 repetitions)
