# Supplementary figures and images for: Phylogenetics, Molecular Species Delimitation and Geometric Morphometrics of All Reddish-Brown Species in the Genus Neotriplax Lewis, 1887 (Coleoptera: Erotylidae: Tritomini)
Source: Insects. 2024 Jul 6;15(7):508. doi: 10.3390/insects15070508 (PMC11277550; doi:10.3390/insects15070508)

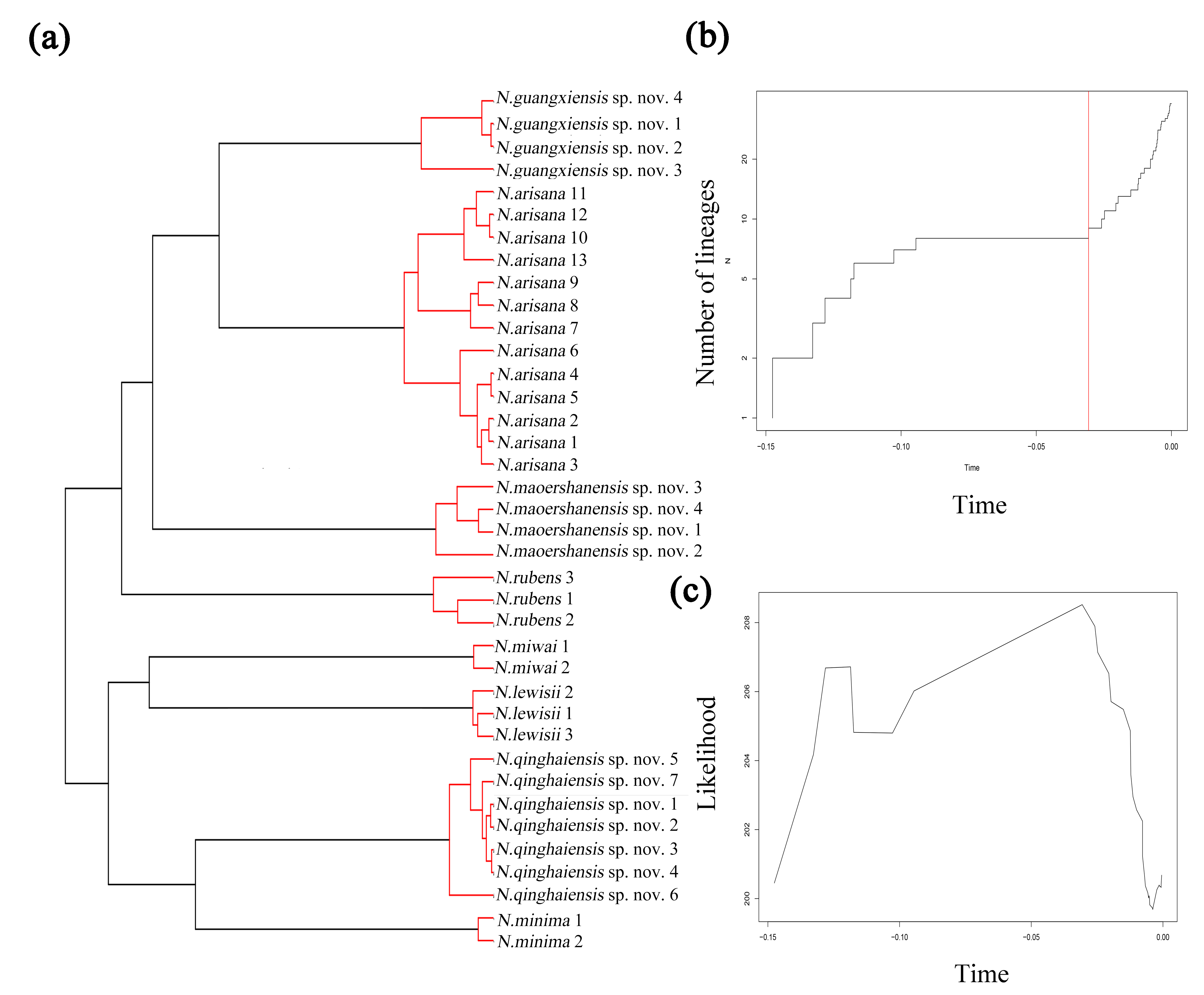

Supplement: Supplementary file 1 [file insects-15-00508-s001.zip › Fig S2. Results of single-threshold GMYC model for Neotriplax based on COI. Species defined by single-threshold GMYC model (a); Relationship between time and lineages, red line.jpg]

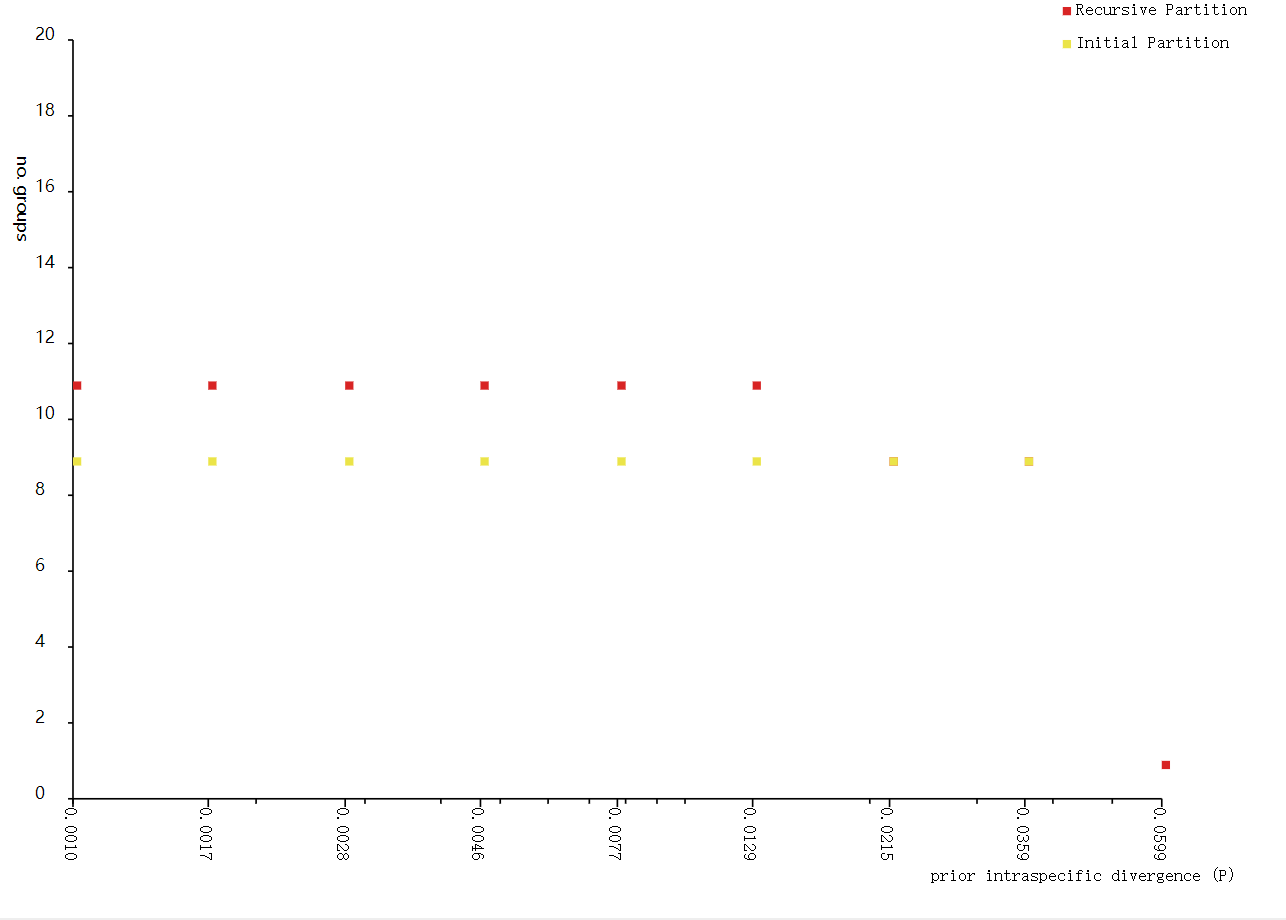

Supplement: Supplementary file 1 [file insects-15-00508-s001.zip › Figure S1.Automatic partition results by ABGD based on COI.png]
